# Focusing on the Neuro-Psycho-Biological and Evolutionary Underpinnings of the Imposter Syndrome

**DOI:** 10.3389/fpsyg.2020.01553

**Published:** 2020-07-20

**Authors:** George P. Chrousos, Alexios-Fotios A. Mentis, Efthimios Dardiotis

**Affiliations:** ^1^University Research Institute of Maternal and Child Health and Precision Medicine, and UNESCO Chair on Adolescent Health Care, “Aghia Sophia” Children's Hospital, National and Kapodistrian University of Athens, Athens, Greece; ^2^Public Health Laboratories, Hellenic Pasteur Institute, Athens, Greece; ^3^Department of Microbiology, School of Medicine, University Hospital of Larissa, University of Thessaly, Larissa, Greece; ^4^Department of Neurology, School of Medicine, University Hospital of Larissa, University of Thessaly, Larissa, Greece

**Keywords:** imposter syndrome, nervous system, neurobiology, stress, hypothalamic-pituitary-adrenal axis, oxytocin

*Oh Tim, I've just had a most ghastly weekend because I felt so unworthy* (Cerejo, 2015). Surprising as it may seem, this quote was directed by Paul Nurse to Tim Hunt shortly after they both learned that they received the Nobel Prize, illustrating, more or less, the so-called *Imposter Phenomenon* (or *Imposter* Syndrome). Originally described in 1978 and increasingly discussed over the last years (with over half of all publications in the area published in the last 6 years), the *imposter syndrome* is a psychological state, in which people express self-doubt on their accomplishments and skills, despite factual evidence or other people indicating otherwise (Bravata et al., 2020). As a corollary, syndromal imposters suffer from constant fear of being exposed as a fraud (*bluff* ), because they often believe that they have fooled their peers into overrating their abilities and professional competence (Chrousos and Mentis, 2020). As part of a vicious cycle, syndromal imposters feel more prone to failure, may become less productive, and are characterized by insecurity and procrastination (Neureiter and Traut-Mattausch, 2016a,b; Mullangi and Jagsi, 2019). Intriguingly, recent studies suggested that imposter syndrome sufferers should be distinguished into two broad categories: *true imposters* and *strategic syndromal imposters*, based on the degree of self-doubt (Leonhardt et al., 2017).

An established body of research has been conducted on the prevalence and consequences of the imposter syndrome (Parkman, 2016; Bravata et al., 2020). Notably, it affects up to two out of three people in certain settings (Gravois, 2007); however, in settings such as academia, its prevalence might be grossly underestimated in the predominant *culture of silence* in higher education (Evans et al., 2018). Highly demanding families and professional environments, psychological traits, such as perfectionism or insecurity, and social inequalities, are all putative contributors to the imposter syndrome (Want and Kleitman, 2006; Dickerson, 2019; Mullangi and Jagsi, 2019; Chrousos and Mentis, 2020). Despite its high prevalence and human toll, the potential neurobiological underpinnings of the syndrome and its evolutionary origin have been scarcely explored.

Here, we emphasize the need to search for the potential psycho-neuro-biological basis and evolutionary roots of this phenomenon, and to highlight the critical research questions that should be addressed and answered in order to change clinical thought and practice. This knowledge will hopefully stimulate novel conceptual approaches to the phenomenon, which may unravel, in the long term, rational methods for its prevention, diagnosis and behavioral -cognitive therapy treatment, especially in the younger generation, especially females, which appear the most afflicted.

In evolutionary terms, it is worth exploring whether the *syndromal imposter phenomenon* could be perceived as a contemporary remnant of a selected trait that could have offered a survival and reproductive advantage to our ancestors, most likely as a result of universal selection within a social hierarchy structure. Specifically, it would be crucial to determine to what extent this phenomenon is a manifestation of *anticipatory* anxiety, a vestige of a primitive arousal status in the anticipation of potential threats (i.e., part of the active *fight-or-flight response*), or of withdrawal from a potential threat, a valuable response of reducing exposure in the face of danger (i.e, a manifestation of the defensive *flight* or passive *freezing response*), or, perhaps, a combination of the two (Chrousos, 2009). In the same vein, could a psychological (not evolutionary) self-induced feeling of failure serve as an internal impetus to reach higher levels of perfectionism, as part of the obsessive-compulsive comorbid traits that are present, more or less, in individuals afflicted by the syndrome? Collectively, could the fear of failure combined with imposter syndrome be associated with the primary emotion of shame, which may have an evolutionary function in keeping the *tribe* together (Jaffe et al., 2014)? Or, at least, in theory, could imposter syndrome sufferers fear success, in which case links to subclinical anhedonia or alexithymia (i.e., difficulty to express feeling of pleasure from success) can be proposed?

Moreover, higher levels of perfectionism or fear of failure or success could make underestimating one's own abilities an evolutionarily stable strategy. For instance, and in a converse manner, could the imposter phenomenon be, as some experts have suggested, a self-deceiving technique aiming to lower anxiety during external judgment of someone's performance, despite internal self-confidence (Young, 2019)? In this context, there might be overlaps of imposter syndrome and cognitive distortions (i.e., dysfunctional though patterns) in various forms of anxiety or depression, especially in relation to the self, often discussed in the context of evolutionary psychiatry (Nettle, 2004). In the same vein, could this syndrome represent a cognitively intense, yet self-harming manifestation of the *premeditatio mallorum* (i.e., negative visualization, or *the pre-meditation of evils*), a concept otherwise defined by Stoic philosophers as a way to reach existential sobriety (Robertson, 2019)?

Importantly, both anxiety and depression are associated with changes in the activity of the stress system, which may lead to chronic brain neuromediator imbalances associated with dysphoric distress (Chrousos, 2009). Pursuing this issue from a neurobiological standpoint, research should focus on whether stress associated with the *imposter syndrome* is linked to elevated release of stress mediators in the Central Nervous System (CNS) and the peripheral tissues of the organism. Stress normally is associated with activation of the brainstem locus caeruleus/norepinephrine system, which subserves arousal and regulates the functions of the autonomic nervous system, and the hypothalamic-pituitary-adrenal (HPA) axis, which plays modulatory roles (Chrousos, 2009). Mediators involved include norepinephrine and corticotropin-releasing hormone in the CNS, and the catecholamines norepinephrine and epinephrine, as well as, the steroid hormone cortisol, in the systemic circulation. Chronic activation of the stress system may be associated with dysphoria, anxiety, and depressive symptoms, as well as, somatization phenomena (Iob et al., 2020); therefore, stress system dysfunction could be linked to some of the most severe mental and somatic manifestations of syndromal imposters.

That said, considering the HPA axis as the biological basis of, or at least a major contributor to, the imposter syndrome should be treated with caution, given that this system is also generally related to stress, anxiety, depression, and somatization, and many other related psychopathologies. Thus, the specificity of the HPA axis in the pathophysiology of the imposter syndrome should not be taken for granted, not least because the predictive power of HPA axis dysfunction for the imposter syndrome might be quite low. Moreover, imposter syndrome and anxiety disorder could activate the same stress response network, thus, raising the question whether they are separate entities or, instead, overlapping ones. Our educated guess is that a major overlap with often comorbid anxiety and depression might be present in the imposter syndrome.

Persons with low self-confidence or those who live in the certainty that others mistakenly overestimate them on a permanent basis, and, thus, constantly fear exposure, should suffer from the burden of the imposter syndrome to a higher degree. Under this prism, the chronically elevated psychological stress level [e.g., as assessed through the Trier Social Stress Test (Kirschbaum et al., 1993)] could be the consequence of the imposter syndrome or, at least, a part of a vicious cycle of cause-causality, with regard to this syndrome; however, examining possible causal relations (or providing suggestions on how to address such causal schemes) would extend beyond the scope of this *Opinion* article. Collectively, it seems imperative, at least in the decades-to-follow, to elucidate the neurobiological basis of the imposter syndrome, to diagnostically and therapeutically approach and its co-morbid disorders in a patient-centered manner toward relieving the discomfort, no matter the underlying pathologic interconnections and causal schemes.

It is noteworthy, nonetheless, that other biological systems, such as the Hypothalamic-Pituitary-Gonadal axis and the associated sex hormones, could be involved in the pathogenesis of the imposter syndrome as well, let alone explaining, at least partially and beyond social and psychological reasons, the increased prevalence of the syndrome in women and girls. Indeed, high testosterone levels have been associated with hierarchy, social dominance, and competition, while estrogen has been connected to agreeableness and cooperation; these effects, which are closely linked to the self and social behavior, may in turn play a role in the genesis of the imposter syndrome (Ehrenkranz et al., 1974; Mccarthy, 1995).

Therefore, the following question has arisen: can we explain and possibly treat the imposter syndrome by targeting the stress system and certain stress mediators? Further research should be conducted, notably by assessing the levels of these mediators in *imposter syndrome* sufferers. Neuro-imaging studies, which, to our knowledge, have not been conducted in affected subjects as yet, would complement the above research efforts.

From a psychobiological perspective, the perceived *effort-reward imbalance* stress model suggests that a disparity between *effort*, which relates to duties and obligations, and *reward*, which includes social and/or financial rewards, can lead to chronic activation of the stress system with all that this entails (Siegrist, 1996; Ota et al., 2014). We are tempted to theorize that *imposter syndrome* sufferers might subjectively perceive *effort-reward imbalance*, which could inhibit them from feeling rewarded for their successes or failing to be optimistic for any kind of upcoming rewards in the future.

In the same context, the role of disturbed levels of stress mediators in contributing to the link between the *imposter syndrome* and *effort-reward imbalance* cannot be excluded, as examined elsewhere (Ota et al., 2014). It is quite plausible that stress mediators, which are either elevated or decreased as a result of chronic stress (Chrousos, 2009), suppress, or fail to activate, the reward system of the brain. For the time being, we contend that in the quest of achieving happiness through success, modern society stimulates activities that ultimately boost the brain reward system, which, however, is known to exhibit tolerance. Thus, on the one hand, intermittently secreted mediators, such as serotonin, oxytocin, and dopamine, the so-called *happiness, affiliation/altruism/compassion, and reward* hormones, respectively, participate in achieving constitutional happiness and optimism, while, on the other hand, continuous or very frequent activation of the stress system could lead to desensitization of the reward system and constitutional unhappiness and pessimism about the future.

Similarly, a chronic decrease in the production of stress mediators, which characterizes some chronic stress states, could cause an inadequate activation of the reward system also accompanied by concurrent dysphoria and pessimism about the future (Chrousos, 2009). In this context, exploring the serotonin, oxytocin, and dopamine systems among imposterism sufferers could be a worthy research pursuit. Likewise, it would be intriguing to know how these neuro-hormones and other bioactive molecules are functionally interconnected (Quintana et al., 2019), and how they are related to the *feelings* of self-doubt in the syndromal imposters.

Could the imposter syndrome be explained and tackled, if its evolutionary and psycho-neuro-biological underpinnings are deciphered? This scientific *journey* may be long-lasting and may entail associating complex psychobiological, neurochemical, and neuroimaging experiments, with scores in imposter syndrome scales [for a review on these scales, see Mak et al. (2019)]. For the time being, the syndromal imposter phenomenon should be named and acknowledged by those who suffer from it, and it should be tackled by a sufferer's realistically appreciation of his/ her own strengths and weaknesses, not by being influenced from misperceived internal or external judgments.

Receiving proper mentoring, seeking the help of experts, and embarking on appropriate psychotherapies may be worthy avenues to pursue in this prevalent syndrome (Chrousos and Mentis, 2020). The latter approaches are of particular importance for the younger generation of high-achievers, because, of note, the age of the majority of PhD and MD-PhD students overlaps with the peak of mental health issue prevalences. These approaches can also be applied to the treatment of broader clinical conditions (such as anxiety disorders), as well as issues of social stigma, thus, offering a holistic approach to the discomfort of imposter syndrome–anxiety sufferers. Last, but not least, in our highly competitive era, one should openly acknowledge that toning down our expectations concerning our own goals to justify potential failures may create vicious cycles of self-discomfort, at least in the so-called *true syndromal imposters* (Leary et al., 2000). The ancient approach of *ethos, pathos*, and *pragma*, i.e., the ethically-, emotionally-, and realistically-oriented approach to life, continues to be pertinent, including suffering from impostor syndrome, and it should always be a worthy salutary goal to pursue.

## Author Contributions

GC, A-FM, and ED conceived and designed the study, prepared the first draft, and reviewed the final version of the manuscript. All authors have read and approved the final version of the manuscript.

## Conflict of Interest

The authors declare that the research was conducted in the absence of any commercial or financial relationships that could be construed as a potential conflict of interest.
